# Stiffness-mediated paracrine signaling enhances induction of EMT in oral squamous cell carcinoma

**DOI:** 10.1063/5.0299456

**Published:** 2025-12-23

**Authors:** So Youn Moon, Marcelo Lazzaron Lamers, Adam J. Engler

**Affiliations:** 1 Chien-Lay Department of Bioengineering, University of California San Diego, 9500 Gilman Drive, MC0695, La Jolla, California 92093, USA; 2 Sanford Consortium for Regenerative Medicine, 9500 Gilman Drive, MC0695, La Jolla, California 92037, USA; 3 Department of Oral Pathology, Federal University of Rio Grande do Sul, Rua Sarmento Leite, 500, subsolo, Porto Alegre, Rio Grande do Sul, Brazil; 4 Deparment of Morphological Sciences, Institute of Basic Health Sciences, Federal University of Rio Grande do Sul, Rua Sarmento Leite, 500, subsolo, Porto Alegre, Rio Grande do Sul, Brazil

## Abstract

Oral Squamous Cell Carcinoma (OSCC) contains diverse communities of cells within the oral mucosa. A subset of the epithelia is highly responsive to changing niche conditions, resulting in their loss of polarity, epithelial-to-mesenchymal transition (EMT), and invasion in tumor-adjacent stroma. Given the range of cell states, we sought to understand how cytokine-mediated signaling from mesenchymal SCC25 cells or stiffness-induced mesenchymal (simCal27) cells caused EMT in naïve Cal27 epithelial cells. Media conditioned by SCC25 enhanced Cal27 cell migration, nuclear localization of EMT markers, and caused transcriptomic changes related to cytokine response ontological terms. SCC and simCal27 cells have unique cytokine profiles, which when regressed against transcriptomic changes, suggested that higher expression of IL-1a, IL-6, IL-8, Angiogenin, and PAI-1 in conditioned media could drive EMT; upregulation of these cytokines also appears impactful for overall survival and progression-free interval. However, depletion and supplementation assays clearly show that the presence of these specific cytokines is critical to induce a migratory phenotype and that naïve Cal27's motility is regulated by MAPK and AKT signaling pathways; loss or inhibition of these pathways reduced migration. These data suggest that paracrine signals from stiffness-induced mesenchymal cells act via distinct kinase pathways and may be necessary for cooperative dissemination of OSCC.

## INTRODUCTION

Oral Squamous Cell Carcinoma (OSCC) accounts for nearly 90% of all metastatic oral cancers.^1^ It evolves from oral lesions that are characterized by dysplastic regions that have irregular rigid margins^2,3^ and significant genetic heterogeneity.^4^ Such variability leads to significant variance in the median 5-year survival, ranging from 40% to 63%.^5^ Such high morbidity^6^ also indicates the need to consider patient-specific aspects of oral tumor biology; variable tumor composition is due in part to regional differences in niche properties and cell signaling that can lead to cells undergoing epithelial-to-mesenchymal transition (EMT),^7^ which is the process where cells lose expression of epithelial (E-Cadherin) but gain mesenchymal markers (N-Cadherin). Cells lose their polarity with EMT, allowing them to increase their motility,^8^ exit the tumor, and recruit other oral cells such that they collectively migrate into tumor-adjacent stroma.^9^

As noted above, EMT and collective migration can be induced by insoluble factors, which include extracellular matrix (ECM) organization and stiffness. Stiffened ECM is one of the ubiquitous non-cellular characteristics of the tumor microenvironment,^10,11^ and stiffness-induced EMT has been studied extensively in many cancer types, e.g., breast, prostate, lung, etc.^10,12–14^ Investigations into this process in OSCC have only begun recently.^15–22^ We previously showed that in stiffer niche, oral epithelia demonstrate aspects of EMT, e.g., increased contractility, N-cadherin expression, and higher motility,^16^ and once they have migrated to softer tumor-adjacent stroma, they can maintain their mesenchymal state via “mechanical memory.^22^” Separately, EMT can also be orchestrated by cytokine networks—i.e., “oncogenic cytokines” such as IL-6 and IL-8—which not only induce EMT but also stimulate proliferation in OSCC;^23^ indeed, expression of IL-6, IL-8, IL-1α, and Tissue Necrosis Factor-α are significantly elevated in both saliva and tissue of patients with OSCC.^24^ While both of these niche parameters have been assessed separately *in vitro*, their cooperative signaling to drive migration and invasion is less clear but likely occurs *in vivo*. In breast and prostate adenocarcinoma, Carey *et al.*^25^ demonstrated such cooperativity in that malignant cells readily invaded ECM and recruited epithelial cells undergoing EMT to follow them. Similar cooperative signaling is present in glioblastoma^26^ and triple negative breast cancer,^27^ and stiffness responses can be modulated through oncogenic cytokines from resident immune cells.^28^

What remains uncertain in OSCC, however, is whether cells exhibiting stiffness-induced EMT are able to “educate” their heterotypic neighbors and cooperatively migrate and invade tumor-adjacent stroma. Hence, we aimed to delineate how cytokine-rich conditioned media from mesenchymal-like (SCC-25) or stiffness-induced mesenchymal (simCal27) cell variants provoke EMT in naïve Cal27 epithelial cells. Mesenchymal-conditioned media harbor elevated IL-1α, IL-6, IL-8, Angiogenin, and PAI-1 levels, which significantly enhanced Cal27 motility. These cytokines, when assessed via regression against transcriptomic changes, implicate immune response and cytokine ontologies, and correlate with poor overall and progression-free survival. Downstream signaling cascades, notably MAPK and AKT pathways, are implicated in mediating cytokine-driven EMT. Taken together, these findings spotlight a dynamic interplay: paracrine signals from mesenchymal-transformed OSCC cells may act through distinct kinase pathways to effectuate EMT and facilitate cooperative invasion.

## RESULTS

### Paracrine signaling between epithelial and mesenchymal cell populations induces phenotype plasticity

**FIG. 1.**
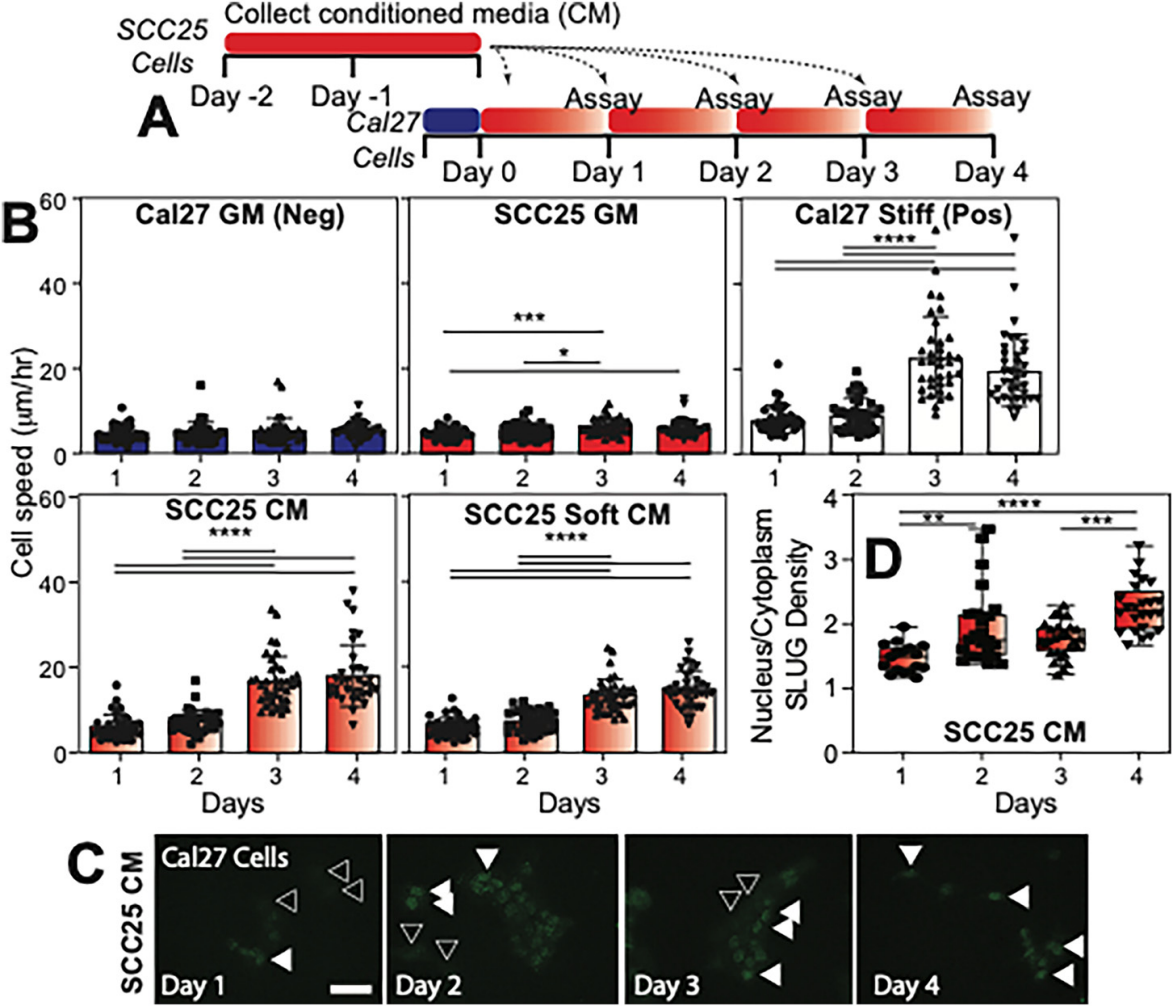
Cal27 migration speed increases after indirect co-culture with SCC25 conditioned media (CM) similar to mechanical memory. (a) Experimental design of SCC25-CM preparation and indirect co-culture of Cal27 with SCC25 using the conditioned media. Cal27 cell speed was measured daily for 4 days after the introduction of conditioned media. (b) Cal27 cell speed was reported after culture in normal Cal27 growth media (blue), SCC25 growth media (red), SCC25 conditioned media (red-to-white), SCC25 conditioned media when the SCC25 cells were grown on a 0.48 kPa soft hydrogel (red-to-white), and when Cal27 cells were cultured on a 20 kPa stiff hydrogel (white; i.e., mechanical memory). (c) Example images of Cal27 cells cultured in SCC25 CM over 4 days and stained for SLUG. Some cells lacking nuclear localization of SLUG are indicated with open arrowheads, while some cells with nuclear localized SLUG are indicated with filled arrowheads. (d) Quantification of the nuclear-to-cytoplasmic ratio of SLUG over time from images in panel (c). ^*^p < 0.05, ^**^p < 0.01, ^***^p < 0.001, and ^****^p < 0.0001 for all data as analyzed by one-way ANOVA with Tukey's multiple comparison test.

OSCC is characterized by significant tumor heterogeneity^29^ and cellular crosstalk, so to understand how cell–cell signaling within the tumor mass can impact naïve epithelial cells (Cal27), we exposed them to conditioned media from mesenchymal cells (SCC25) and compared media vs stiffness-induced mesenchymal cells (simCal27)^22^ [Fig. 1(a)]. Cal27 cells in any unconditioned media did not exhibit signs of EMT, but in media conditioned by SCC25 cells, Cal27 cells migrated faster by day 3 [Fig. 1(b)] and exhibited signs of EMT with SLUG nuclear localization [Figs. 1(c) and 1(d)]. Importantly, this effect was independent of matrix stiffness for the inducing cells, i.e., the SCC25 cells conditioning media while being cultured on soft matrix or not. It is also important to note that media-based induction resulted in motility and time course similar to simCal27 cells [white vs gradient bars, Fig. 1(b)]. Thus, both culture condition and time appeared to play statistically significant roles in EMT in producing cell responses similar to simCal27 cells (Fig. S1).

### A small cytokine profile is necessary to drive EMT in oral epithelia

**FIG. 2.**
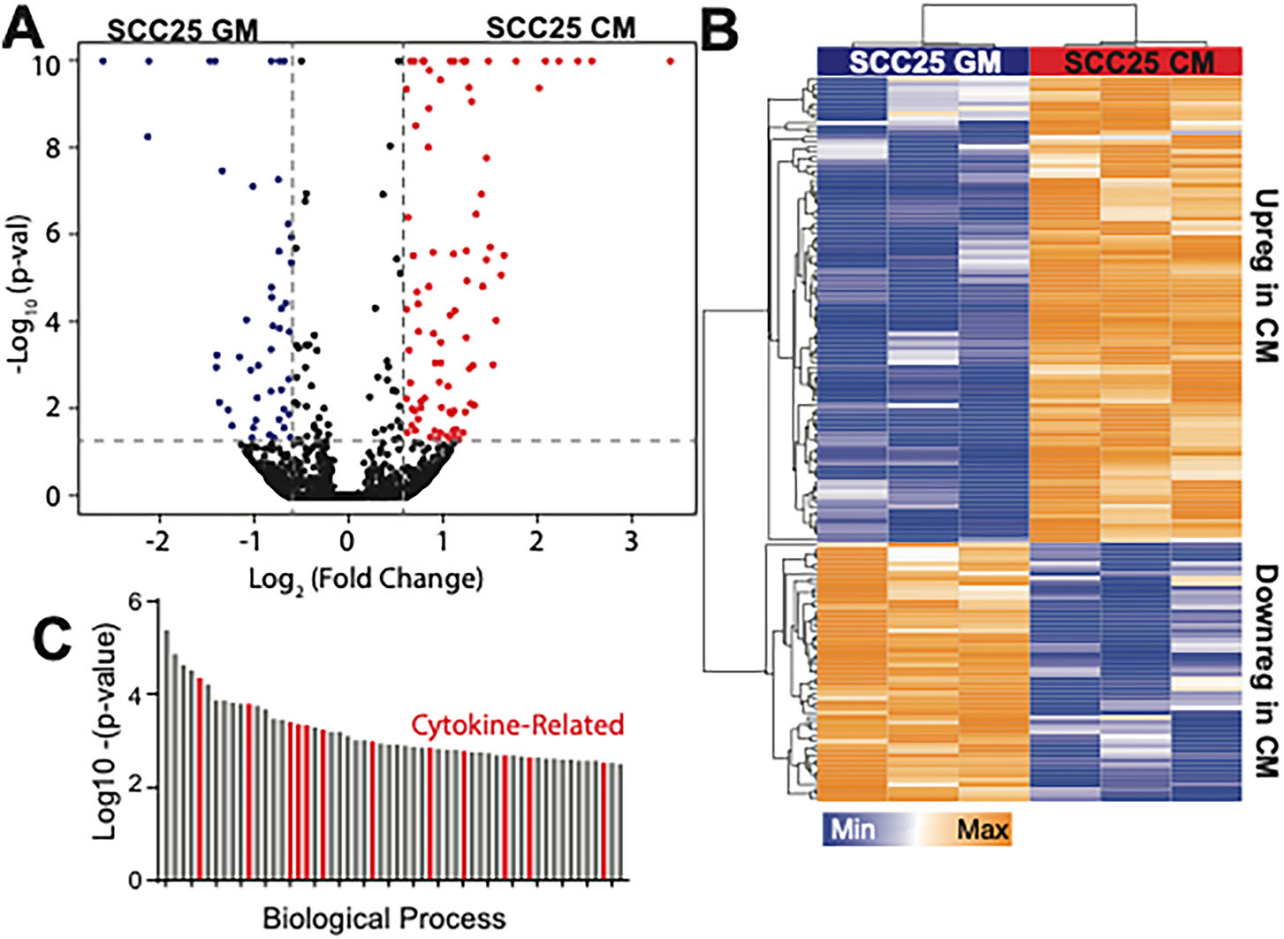
Transcriptomic change in Cal27 after being cultured with SCC25-CM for 3 days. Volcano plot (a) and heat map (b) of the genes from bulk RNA sequencing comparing Cal27 cultured with SCC25-CM for 3 days (red) vs with SCC25-GM for a day (blue). DEGs in the volcano plot were filtered with log_2_-fold change of >0.15 (up-regulated) or <−0.15 (down-regulated) and −log10(p-value) > 1. Hierarchical clustering of DEGs is shown in the heat map. (C) Biological process Gene Ontology (GO) terms based on DEGs. The terms related to cytokine response or cell motility change are marked in red.

**FIG. 3.**
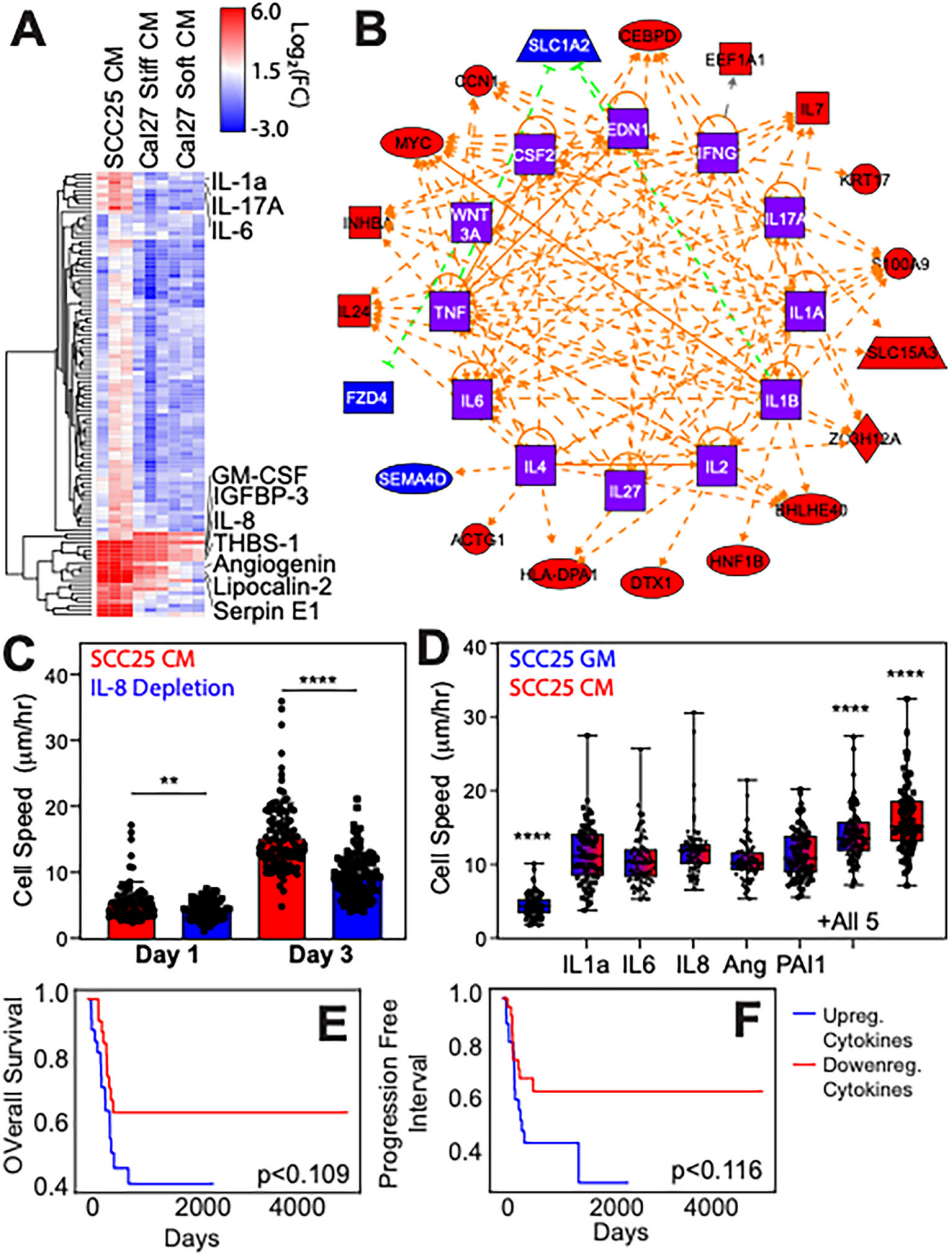
Cytokines involved in metastasis-inducing paracrine cell signaling. (a) Heatmap of cytokine arrays measuring the expression of 105 cytokines from culture media conditioned by SCC25 cells, Cal27 cells on stiff 20 kPa hydrogels, and Cal27 cells on soft 0.5 kPa hydrogels. Data were normalized to fresh growth media. Red and blue indicate up and down-regulation of each cytokine (fold-change) relative to fresh growth media. (b) Upstream regulator analysis map from QIAGEN Ingenuity Pathway Analysis (IPA). From up (red) or down (blue) regulation of genes from RNA seq result, possible upstream regulators (orange) were extrapolated. Orange and green arrows indicate predicted activation and inhibition between two components, respectively. Square refers to cytokine, circle is complex/group, rhombus is enzyme, rectangle is G-protein coupled receptor, horizontal oval is transcriptional regulator or transmembrane receptor, and trapezoid is transporter. (c) Cell speed for Cal27 cells cultured with media conditioned by SCC25 cells and selectively depleted of IL-8. Speed was measured on Day 1 and Day 3 of conditioned media incubation. (d) Fresh SCC25 growth media (blue) was supplemented with 1 ng/ml of cytokines of either IL-1a, IL-6, IL-8, Angiogenin, or PAI-1 (blue-to-red), and Cal27 cell speed was measured at day 3 in culture. Data are compared to Cal27 cells cultured on a 20 kPa stiff hydrogel (red). ^*^p < 0.05, ^**^p < 0.01, ^***^p < 0.001, and ^****^p < 0.0001 for all data as analyzed by one-way ANOVA with Tukey's multiple comparison test. Cytokines filtered and tested from panels (a) and (b) were used to stratify patients in The Cancer Genome Atlas (TCGA). The top and bottom quantiles of patients in terms of transcriptomic-level cytokine expression had their overall survival (e) and progression-free interval (f) compared. The significance of the difference between two groups was analyzed with a log-rank (Mantel–Cox) test.

While nuclear localization is a key indicator of EMT, we more broadly assessed transcriptomic changes to Cal27 cells in the presence of SCC25 conditioned media at day 3. Using media conditioned from mesenchymal cells (SCC25), naïve epithelial Cal27 cells had 151 differentially expressed genes (DEGs) [Figs. 2(a) and 2(b); Table S1]. Gene Ontology (GO) terms were mapped onto these DEGs, and we found a mix of cellular component terms associated with epithelial and mesenchymal phenotypes (Table S2, yellow vs orange, respectively), suggesting that paracrine signals, whether coming from mesenchymal or simCal27 cells, may create a partially induced, transitional state^30^ that may vary over time.^31^ For biological processes ontology terms, we found a dozen associated with cellular responses to cytokine and intra-cellular signaling pathways [Fig. 2(c), red; Table S3, yellow]. To more directly identify the cytokines responsible for Cal27 EMT, we studied the cytokine profile of both SCC25 and simCal27 conditioned media relative to media conditioned by naïve Cal27 cells. Only one cytokine, GDF-15, was upregulated in naïve epithelial-conditioned media. However, seven cytokines were upregulated in both SCC25 and simCal27 media relative to naïve epithelial-conditioned media: GM-CSF, IGFBP-3, IL-8, Thrombospondin-1, Angiogenin, Lipocalin-2, and Serpin E1 [Fig. 3(a), red; Table S4]. To create a more inclusive list of stiffness-induced mesenchymal Cal27 cytokines, DEGs from sequencing were included in an Ingenuity Pathway Analysis (IPA) map to predict upstream regulators that are likely to be activated or inhibited in both SCC25 and simCal27 media. Among upstream regulators identified by IPA [Fig. 3(b)], three cytokines were upregulated in SCC25 cytokine array only, i.e., IL-1a, IL-17A, and IL-6, while most cytokines upregulated in both SCC25 and simCal27 cells were also present in the network. These ten cytokines were further verified with enzyme-linked immunosorbent assay (ELISA) (Fig. S2). The concentrations of GM-CSF, IGFBP-3, Thrombospondin-1, Lipocalin-2, and IL-17A were similar or higher for naïve Cal27 cells vs SCC25. However, five cytokines, i.e., IL-1a, IL-6, IL-8, Angiogenin, and PAI-1 (Serpin E1), agreed between arrays and ELISAs where media conditioned by mesenchymal cells was higher.

For functional validation, only IL-8 could be immunoprecipitated from conditioned media [Fig. S2(c)]; we found that naïve Cal27 cells had reduced cell migration speed in IL-8-depleted conditioned media, independent of time, relative to IL-8 containing conditioned media [Fig. 3(c)], suggesting that IL-8 is necessary to enhance cell migration similar to mesenchymal cells. To understand the degree to which the other four cytokines could regulate migration, we supplemented unconditioned media with each cytokine individually. However, we first titrated the concentration of all five cytokines to identify a viable concentration that enhances cell motility similar to conditioned media, and 1 ng/mL appeared sufficient (Fig. S3). When each cytokine was individually added to unconditioned media, we found that all cytokines equally increased naïve Cal27 cell motility, and when combined, their effects were additive [Fig. 3(d)].

Expression of IL-1a, IL-6, IL-8, Angiogenin, and Serpin E1 was then used to stratify patients' bulk RNA sequencing data from The Cancer Genome Atlas (TCGA). 155 head–neck squamous carcinoma (HNSC) patients whose primary tumor site was either the tongue or unspecified parts of the tongue were computed. Overall survival (OS) [Fig. 3(e)] and progression-free interval (PFI) [Fig. 3(f)] of the top and bottom quantiles showed noticeable differences, suggesting that these few cytokines from SCC25 and simCal27 cells can locally drive EMT in naïve oral epithelia but may globally impact patient trajectories.

### Cytokine stimulation induces MAPK and AKT signaling in naïve oral epithelia

**FIG. 4.**
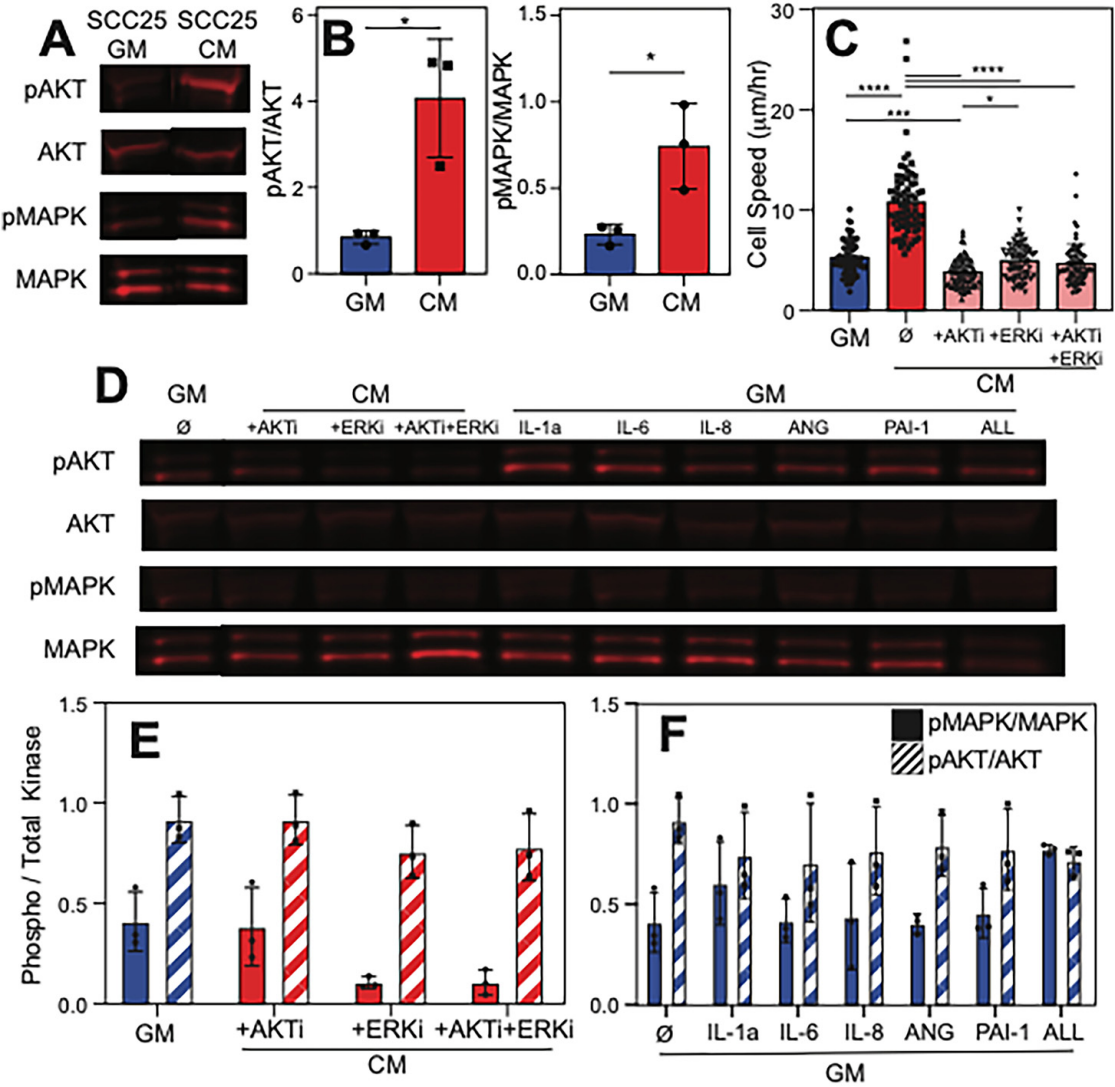
MAPK and AKT signaling pathways activated in response to paracrine cell signaling. (a) Western blot image of phosphorylated/total AKT and MAPK (ERK1/2) in Cal27 cells cultured with normal SCC25 growth media or conditioned media. (b) Quantification of phosphorylated to total kinase expression from western blots in panel (a) of AKT (left) and MAPK (right). (c) Cell speed was plotted for Cal27 cells cultured in SCC25 growth media (blue) or conditioned media (red) and AKT and/or MAPK with inhibitors (pink). (d) Western blot of Cal27 cells cultured with SCC25 growth media (left), conditioned media with AKT and/or MAPK with inhibitors as indicated (center), or growth media with individual or all human recombinant cytokines added as indicated (right). (e) and (f) Quantification of western blot result from (d). ^*^p < 0.05, ^**^p < 0.01, ^***^p < 0.001, and ^****^p < 0.0001 for the data as analyzed by one-way ANOVA with Tukey's multiple comparison test.

To understand how the cytokines from stiffness-induced mesenchymal cells stimulate EMT in naïve oral epithelial cells, we performed targeted pathway inhibition in the presence of conditioned media. Since all five cytokines above increase the migration speed of naïve cells, we focused on MAPK and AKT pathways as they are commonly related to migration^32–34^ and “cellular memory.^22^” ERK1/2 and AKT were both significantly phosphorylated in naïve Cal27 cells when cultured in media conditioned by mesenchymal cells [Figs. 4(a) and 4(b)]. Inhibition of either or both pathways via Perifosine (AKT inhibitor) and/or SCH772984 (ERK 1/2 inhibitor) significantly reduced the ability of conditioned media to increase migration speed of naïve Cal27 cells [Fig. 4(c)]. To validate that reduced migration was the result of the appropriate pathway's inhibition, we again examined pathway phosphorylation by western blot [Fig. 4(d)]. Inhibition resulted in reduced ERK1/2 phosphorylation and no change in off-target or inhibitor controls. However, AKT signaling remained unaffected [Fig. 4(e)] despite migration changes, suggesting that the MAPK pathway is primarily activated during paracrine signaling between mesenchymal and naïve oral epithelial cells. Addition of each individual cytokine showed similar ERK1/2 and AKT responses to controls [Fig. 4(f)], which also suggests that smaller increases in migration speed observed above might be amplified once MAPK signaling cascades kick in when additional cytokine signals are present.

## DISCUSSION

Given the large cellular community and wide range of niche within the oral mucosa, it is critical to understand how these disparate cells communicate, what drives them, and what are the consequences of their communication. Cells disseminating from OSCC typically migrate collectively into tumor-adjacent stroma,^9^ meaning that cell–cell communication during stromal invasion is critical to successful metastasis. As we have previously shown, stiffened ECM can induce EMT in oral epithelia,^16^ and their “mechanical memory” can maintain their stiffness-induced mesenchymal (simCal27) phenotype once in softer matrix.^22^ Mesenchymal cells must use paracrine signals to communicate extensively when cooperatively migrating outside of the tumor;^35^ hence, two outstanding questions: (1) can mesenchymal cells “educate” their heterotypic neighbors, and (2) can SCC25 and simCal27 cells signal to naïve epithelial cells similarly to established mesenchymal cells? Data suggested that conditioned media from both SCC25 and simCal27 cells are able to provoke EMT and migration changes in naïve Cal27 cells, implying that simCal27 cells express a mesenchymal cytokine profile like their SCC25 counterparts. Moreover, both cell lines express a defined set of cytokines—IL-1α, IL-6, IL-8, Angiogenin, and Serpin E1—that activate MAPK and AKT signaling, enhance motility, and correlate with poor patient outcomes.

### EMT is cooperative and requires soluble and insoluble components of the OSCC tumor microenvironment

Growing evidence suggests that OSCC progression relies on both cell-autonomous mechanisms and non-cell-autonomous interactions between heterogeneous tumor subclones;^36–38^ however, data here highlight how cooperative migration may occur from a tumor, i.e., stiffness changes that induce EMT in select cells continue to cancerize the local environment through cytokine secretion, thereby converting neighboring epithelial cells into a range of motile, invasive phenotypes depending on the degree of their transition, as seen elsewhere.^30^ Cell states remain dynamic^31^ but result in cooperative behavior with parallels in breast, ovarian, and prostate cancers where invasive “leader” cells recruit less motile neighbors during collective invasion.^39–41^ Similarly in glioblastoma, paracrine signals have been shown to reinforce heterogeneity and invasion dynamics.^42^ Here, data suggest this paradigm to OSCC, underscoring that mesenchymal subpopulations function as both initiators and sustainers of invasion through paracrine “education” of epithelial neighbors with cytokines. Among the cytokines elevated in mesenchymal-conditioned media, IL-1α, IL-6, IL-8, Angiogenin, and PAI-1 emerged as potent inducers of EMT in naïve oral epithelial cells. Each cytokine individually enhanced motility at physiologically relevant concentrations, and their combined effect was additive, suggesting cytokine cooperativity. This observation aligns with prior studies showing that IL-6 and IL-8 are key mediators of cancer progression in OSCC and other epithelial cancers.^23,43^ More specifically, their dual inhibition inhibits tumor cell migration,^44^ and in OSCC, both are key diagnostic markers in saliva^24^ for highly invasive tumors. Though less studied in OSCC, Serpin E1 (PAI-1) and Angiogenin are increasingly recognized as mediators of invasion and poor prognosis in multiple cancers.^45,46^ Thus, the convergence of canonical pro-inflammatory cytokines (IL-1α, IL-6, and IL-8) with less conventional mediators (Serpin E1 and Angiogenin) highlights the multifaceted paracrine programming of EMT in OSCC.

Our findings also intersect with the emerging concept of “mechanical memory” in cancer.^47^ We previously demonstrated that OSCC cells exposed to stiff ECM retain mesenchymal traits even when returned to softer environments.^22^ We have also shown that cancer cells can transmit mechanical behavior via cytokines to another cell population.^48^ The present study connects these two concepts: that mechanically primed cells, i.e., simCal27, can secrete cytokines that propagate EMT to naïve neighbors, effectively transmitting mechanical memory across a population through biochemical means in a cell extrinsic manner. “Mechanical memory” was first shown in breast cancer^27^ and recently shown to transfer their memory by remodeling collagen fibers to promote invasion of adjacent cells.^49^ Although a variety of transfer mechanisms have been proposed,^50^ data here suggest that the oncogenic cytokines mentioned above are used by OSCC to educate adjacent phenotypes. Collectively, stiffness-induced EMT may be both cell-intrinsic and community-enforced, creating a feedback loop between biomechanical and paracrine cues. Multiple mechanisms could also explain the persistence of EMT and collective invasion, even after cells migrate away from stiff tumor cores into softer stromal regions.

Finally, we found that conditioned media from mesenchymal OSCC cells activates both MAPK/ERK and AKT signaling in naïve Cal27 epithelia, which are pathways known to regulate motility, survival, and EMT.^51^ These findings are consistent with reports that IL-6 and IL-8 can activate ERK1/2 and AKT cascades in epithelial tumors, promoting invasion and therapy resistance.^52^ Importantly, our data show that individual cytokines can trigger ERK/AKT activation, but the full effect is realized only in the presence of multiple cytokines, suggesting signal amplification through pathway convergence. This mechanistic insight supports the concept that EMT in OSCC is not driven by isolated signals but by a complex network of redundant and cooperative cues.

### Limitations of in vitro, reductionist models

The tumor microenvironment is complex, especially in OSCC where multiple cell types and fluids form the niche. While reductionist approaches isolate individual tumor components, they eliminate other niche signals that could also modulate the response measured. For example, data here employ conditioned media, which while useful for modeling paracrine signaling, cannot fully recapitulate the spatial and temporal complexity of the tumor microenvironment, where cytokine gradients, ECM context, and cell–cell contacts influence EMT.^53^ Functional validation also focused primarily on migration assays, but EMT encompasses broader phenotypes including invasion, resistance to apoptosis, and stemness, which were not addressed.^8^ That combined with the reliance on Cal27 and SCC25 cell lines may not capture patient variability; primary OSCC cultures and oral epithelial organoid systems have recently become available and could yield more clinically relevant findings.^54^ Although data here were input into *in silico* patient models via TCGA stratification, which suggested prognostic relevance of cytokines, this correlation may not completely establish causality.^37^ Finally, off-target effects from inhibitors, e.g., Perifosine, may interfere with lipid-related metabolism, signal transduction, and membrane function.^55^ Though the most well-known effect of Perifosine is its inhibition of AKT pathway, it can also inhibit other kinases,^56^ even ERK/MAPK.^55,57–59^ Since Perifosine functionally interrupted paracrine-mediated migration, one of the signaling pathways other than AKT that can be inhibited by Perifosine, e.g., ERK as shown in Fig. 4(e), may be involved in the process and should be studied to further understand intra-tumor paracrine cell signaling between heterotypic cell populations in OSCC.

## CONCLUSION

Data here demonstrate that mesenchymal OSCC cells drive EMT in naïve epithelial neighbors through secretion of a limited cytokine set—IL-1α, IL-6, IL-8, Angiogenin, and Serpin E1—which converge on MAPK and AKT signaling pathways to enhance migration and invasion. These cytokines stratify patient outcomes, linking paracrine EMT with clinical progression. Our findings highlight the cooperative nature of OSCC invasion, wherein mechanical cues and paracrine cytokine signaling reinforce one another to sustain tumor heterogeneity and aggressiveness. Collectively, our findings suggest that tumor heterogeneity in OSCC is not only a product of intrinsic genetic variation but is actively sustained by cytokine-mediated cooperative signaling across tumor subpopulations.

## METHOD

### Cell culture and reagents

OSCC cell lines—Cal27 and SCC25—were a kind gift from Akihiro Sakai, University of California San Diego. Cal27 cells were cultured in DMEM high glucose media supplemented with 10% fetal bovine serum (FBS) and 1% penicillin/streptomycin (i.e., Cal27-GM). SCC25 cells were cultured in 1:1 DMEM and F12 with HEPES supplemented with 10% FBS, 1% penicillin/streptomycin, and 400 ng/ml of hydrocortisone (Sigma; SCC25-GM). For the media control group in the indirect co-culturing assay, Cal27 was cultured with cell culture media prepared for SCC25 (1:1 DMEM/F12 with 10% FBS, 1% penicillin/streptomycin, and 400 ng/ml hydrocortisone) to support that the base media difference does not affect cell behavior. Cells were treated with recombinant human proteins to see how cytokines of our interest functionally affect cells. IL-1a, IL-6, IL-8, and Angiogenin were obtained from R&D System, and PAI-1 was obtained from Pepro Tech. Cells were treated with 1, 10, and 100 ng/ml of each cytokine in the time-lapse assay to check the effectiveness of individual cytokines. Cells were treated with each recombinant human protein in 1:1 DMEM/F12 (10% FBS, 1% penicillin/streptomycin, and 400 ng/ml hydrocortisone). 5 *μ*M Perifosine (MedChemExpress) and 4 nM SCH772984 (SelleckChem) were selectively added to cultures to inhibit AKT and ERK, respectively. All cell culture reagents were acquired from Thermo Fisher unless otherwise noted.

### Polyacrylamide hydrogels

The glass surface was oxidized by UV/ozone exposure (BioForce Nanosciences) and functionalized with 20 mM 3-(trimethoxysilyl)propyl methacrylate (Sigma-Aldrich) in ethanol. 0.1% (w/v) ammonium persulfate (APS) (Thermo Fisher) and 0.1% (v/v) N,N,N′,N′-tetramethyl-ethylenediamine (TEMED) (VWR) were added to (w/v) 3% acrylamide and 0.06% bis-acrylamide solution in PBS for 0.48 kPa polyacrylamide gel, or 8% of acrylamide and 0.264% of bis-acrylamide for 20 kPa. 30 *μ*l of pre-PA solution for a 12-well glass bottom tissue culture plate (Corning) and 40 *μ*l for 25 mm coverslips were sandwiched between a methacrylate functionalized glass surface and dichlorodimethylsilane (DCDMS)-treated (Thermo Fisher) coverslips or glass slides. After 10 (for 20 kPa PA) or 30 (for 0.48 kPa) minutes of incubation at room temperature, DCDMS-treated coverslips/glass slides were detached. PA gels were incubated with 0.2 mg/ml sulfo-SANPAH (Pierce) in 50 mM HEPES at 350 nm wavelength UV for 10 min. After three times of rinsing with PBS, PA gels were incubated overnight in 0.15 mg/ml collagen type I (company, catalog) in PBS at 37 °C. Collagen-coated gels were UV-sterilized for 45 min before cells were seeded on them.

### Indirect co-culture

To prepare SCC25 conditioned media, 2 × 10^5^ SCC25 cells per well were seeded on either a 6-well plastic tissue culture plate or 0.48 kPa PA gel on a 25 mm coverslip with 2 ml of SCC25 culture media per well. After 24-h incubation at 37 °C and 5% CO_2_, cell cultured media was replaced and further incubated for 24 h. Conditioned media was collected and sterile filtered with a 0.22 *μ*m pore size steriflip (Sigma-Aldrich). Conditioned media was used to culture naïve Cal27 on 0.48 kPa PA gel right after collection without being stored. Cal27 from tissue culture plastic flask were trypsinized and seeded on collagen-coated 0.48 kPa PA gel (every control and experimental groups but positive control) or 20 kPa PA gel (for positive control). After 2 h of incubation at 37 °C and 5% CO_2_, cell culture media was replaced with fresh or conditioned media. The effect of indirect co-culturing was observed for 4 days. To identify cytokines involved in paracrine cell–cell interaction, 1:1 DMEM/F12 media was supplemented with recombinant human proteins instead of being conditioned by SCC25. The Cal27 seeding and incubation process was the same, but 1, 10, and 100 ng/ml of IL-1a, IL-6, IL-8, Angiogenin, and PAI-1 were added to 1:1 DMEM/F12 fresh media for the titration assay. After titration, the concentration of individual cytokines was set to 1 ng/ml.

### Time-lapse video microscopy

After being plated on either 0.48 or 20 kPa PA gels and cultured with appropriate cell culture media for varying duration of time, Cal27 migration was observed with a Nikon TiU inverted phase/fluorescence microscope with a programmable stage, multi-beam, six-color Yokogawa CSU-X1 confocal attachment for imaging at 2000 FPS. The microscope is also outfitted with a temperature and CO_2_ controller (Pathology Devices Inc., LiveCell). Images of cells at 10× magnification in brightfield in multiple positions were taken every 15 min for 20 h. The “Manual Tracking” plugin on ImageJ was used to track each cell. The nucleus of each cell was used as a reference point to track.

### RNA sequencing

RNA from Cal27 cells cultured with SCC25-CM for 3 days vs those cultured with fresh DMEM/F12 1:1 media for a day was collected by RNeasy Mini Kit (QIAGEN, 74104). The Institute for Genomic Medicine at the University of California San Diego processed the extracted RNA. RNA integrity and concentration were analyzed by the Agilent Tape Station system and Qubit 2.0 Fluorometer, respectively. All submitted samples had RIN numbers above 9.0. The RNA library was prepared with Illumina TruSeq Stranded RNA, High Throughput Library Prep Kit, and then sequenced on a NovaSeq 6000. ROSALIND^®^ was used to analyze sequencing data with a HyperScale architecture (ROSALIND, San Diego, CA). ROSALIND trimmed the reads with cutadapt, assessed the quality score with FastQC, and aligned the reads to the human genome build dm6 with STAR. Sample reads were quantified with HTseq. The DESeq2 R library normalized the read count by Relative Log Expression (RLE) and calculated log fold changes and p-values, which are adjusted by optional covariate correction. Clustering of DEGs was visualized on the heatmap generated by the Partitioning Around Medoids method using the fpc R library. Functional enrichment analysis of pathways and gene ontology was done by HOMER. Panther was used to assess GO terms for DEGs.

### Cytokine array and media depletion

Cytokine expression profiles of conditioned media—SCC25-CM, Cal27 stiff-CM, and Cal27 soft-CM—were measured by the Proteome Profiler Human XL Cytokine Array Kit (R&D Systems; ARY022B). 2 × 10^5^ cells of SCC25 on a plastic tissue culture plate, and the same amount of Cal27 were seeded on either a stiff (20 kPa) or soft (0.48 kPa) substrate. Cells were incubated with DMEM/F12 1:1 (10% FBS, 1% penicillin/streptomycin, and 400 ng/ml hydrocortisone) for 24 h. After conditioning, the media was sterilized with steriflip. To detect cytokines from the samples, the cytokine array from the R&D system used a membrane-based sandwich immunoassay. Nitrocellulose membranes pre-coated with 105 different capture antibodies were incubated with conditioned media at 4 °C overnight on a shaker. The membranes were washed three times with washing buffer. The cytokines attached to the capture antibody on the membrane were once more detected by biotinylated detection antibodies. The membranes were further treated with streptavidin–horseradish-peroxidase (HRP) conjugate. Membranes were coated with Chemi Reagent Mix from the kit and developed on x-ray film with autoradiography for 5 min. The array results were analyzed by semi-automated analysis of dot blots with ImageJ. The immunoprecipitation method was used to deplete IL-8 from SCC25-CM. 500 *μ*l of SCC25-CM was combined with 10 *μ*g of anti-IL-8 antibody (R&D Systems, MAB208-100) and mixed overnight at 4 °C. 0.25 mg of Protein A/G Magnetic beads (Pierce, PI88802) were washed twice with TBST buffer (0.2% Tween 20). After washing, the conditioned media and antibody mixture was added to the Eppendorf tube with the beads and mixed for an hour at room temperature. The beads were collected with a magnetic stand, and the flow-through was collected. IL-8-depleted SCC25-CM was sterilized with Steriflip and used for ELISA or cell culture assay without storage.

### Ingenuity pathway analysis (IPA)

Cytokine expression array and RNA-seq data were further analyzed through the use of IPA (QIAGEN Inc., https://www.qiagenbioinformatics.com/products/ingenuitypathway-analysis). GO terms for co-regulated DEGs were identified using Gene Enrichment Analysis powered by PANTHER version 14.

### Enzyme-linked immunosorbent assay

To quantify the cytokine expression in SCC25-CM, and Cal27 on soft-CM, sandwich ELISA was used. ELISA for IL-8 exceptionally had three samples including the IL-8 eliminated version of SCC25-CM with immunoprecipitation. The ELISA kit used for each target is like the following: Angiogenin (abcam, ab219629), GM-CSF (BioLegend, 432004), IGFBP-3 (Invitrogen, EHIGFBP3), IL-1a (abcam, ab178008), IL-17A (abcam, ab216167), IL-6 (abcam, ab178013), IL-8 (BioLegend, 431504), Lipocalin-2 (abcam, ab215541), PAI-1 (abcam, ab269373), and Thrombospondin-1 (Invitrogen, EHTHBS1). Instructions for each kit were followed. The standard of each ELISA run was prepared with diluent in the kit. Microplates were coated with capture antibody (overnight), blocking buffer (1 h), diluted samples (2 h), and detection antibody (1 h) in order (BioLegend and Invitrogen) or all of them (1 h) together (abcam) at room temperature on a plate shaker. In between every step, the plates were washed three times with washing buffer. The detection antibodies are conjugated with biotins. The plates were incubated with streptavidin–HRP solution (30–45 min) and then TMB substrate (5–15 min) at room temperature on a shaker. The reaction of HRP and TMB substrate was stopped with stop solution. The plates' optical density (OD, 450 nm) with reference OD at 570 nm was read with an Envision plate reader (Revvity, 2104-0010) using Tecan software.

### Western blot

Cal27 was seeded on soft PA gel with Cal27-GM and incubated for 2 h. After incubation, the media was replaced with either SCC25-GM, SCC25-GM supplemented with cytokine, or SCC25-CM and incubated 30 min further. The cells were lysed with mRIPA buffer, and the amount of protein in cell lysates was quantified with the BCA assay kit (Thermo Scientifics, 23225). 10 *μ*g of protein per sample was denatured by 4× Laemmli buffer (BioRad, 1610747), 50 mM DTT, and mRIPA. Denatured samples were deactivated at 95 °C for 5 min. The samples were loaded to 4%–12% Bis-Tris gel (Thermo Fisher Scientific, NW04122BOX), and the gel underwent electrophoresis under 70 V. The gel was blotted on a nitrocellulose membrane with an iBlot semi-dry transfer system (Invitrogen, IB301001). The membrane was blocked with Azure Fluorescent Blot Blocking Buffer (Azure Biosystems, AC2190) with 1% bovine serum albumin (BSA) on a shaker at room temperature. After blocking, the membrane was incubated with primary antibody—ERK 1/2 (1:1000, Cell Signaling Technology, #4695), phospho-ERK 1/2 (1:1000, Cell Signaling Technology, #9101), AKT (1:1000, Cell Signaling Technology, #9272), and phospho-AKT (1:1000, Cell Signaling Technology, #4060)—overnight at 4 °C, and then with donkey anti-rabbit IgG secondary antibody (1:2000, 680 nm, Invitrogen, A10043) at room temperature for 1 h on a shaker. In between the steps of antibody coating and before imaging, the membrane was washed (3×) with TBST buffer for 5 min on a shaker. The image was developed with LI-COR Odyssey CLx imaging software and analyzed by Image Studio Lite. The amount of phosphorylated proteins was normalized by the expression of the total amount of the protein.

### The cancer genome atlas dataset analysis

The Cancer Genome Atlas (TCGA) raw data were directly downloaded from the NIH NCI GDC Data portal. Corresponding clinical metadata were obtained from a previous publication. Among the patient data with head–neck squamous carcinoma (HNSC), those whose primary site is either the base of the tongue or other and unspecified parts of the tongue were used for analysis. To correlate the 5-year survival rate of patients with transcriptomic upregulation of cytokines that are likely to be involved in paracrine cell–cell metastasis-inducing communication, we normalized the expression of each gene per patient with a z-score based on the mean of the gene's expression across the whole patient data. Per patient, the normalized z-score of five cytokine genes was summed. Z-score was used to determine the top and bottom quantiles, which were mapped on a Kaplan–Meyer plot in “Cytokines Up-regulated” vs “Cytokines Down-regulated” groups. Overall survival (OS) and progression-free interval (PFI) comparing top and bottom quantiles were plotted with the Kaplan–Meier method. The log-rank test was used to determine the significance of the survival rate difference between two groups. Survival analysis is included in the Lifelines python library (https://lifelines.readthedocs.io/en/latest/).

### Statistics

All data are shown from triplicate biological replicates, with the total number of measurements made indicated in figure legends. For all relevant assays, data were analyzed in triplicate by one-way ANOVA with Tukey's post-test or multiple comparisons test as indicated. For all analyses, ^*^p < 0.05, ^**^p < 0.01, ^***^p < 0.001, and ^****^p< 0.0001. Significance for TCGA analyses was performed with a log-rank (Mantel–Cox) test. All other values are expressed as mean +/− standard deviation. Statistical analyses were performed using Prism software.

## SUPPLEMENTARY MATERIAL

See the supplementary material for the following: plot of two-way ANOVA test for the effect of cell-culture media and culturing time on Cal27 cell speed (Fig. S1); plot of enzyme-linked immunosorbent assay (ELISA) measuring cytokine expression in media conditioned from SCC25 cells, in cytokine-depleted SCC25 conditioned media, and Cal27 conditioned media (Fig. S2); plot of the titration test of human recombinant cytokines (Fig. S3); table of bulk RNA-sequencing results for Cal27 cells cultured in SCC25-CM for 3 days vs in SCC25-GM for 1 day (Table S1); table of cellular component gene ontologies for Cal27 cells cultured in SCC25-CM for 3 days vs in SCC25-GM for 1 day (Table S2); table of biological process gene ontologies for Cal27 cells cultured in SCC25-CM for 3 days vs in SCC25-GM for 1 day (Table S3); and table of the cytokine profile of conditioned media relative to naïve cells (Table S4).

## Data Availability

The data that support the findings of this study are openly available in OSCC_Memory at https://github.com/englea52/OSCC_Memory.git, Ref. 60.
